# Insights into the Bioprospecting of the Endophytic Fungi of the Medicinal Plant *Palicourea rigida* Kunth (Rubiaceae): Detailed Biological Activities

**DOI:** 10.3390/jof7090689

**Published:** 2021-08-25

**Authors:** Igor Romeiro dos Santos, Ahmed M. Abdel-Azeem, Marwa T. Mohesien, Magdalena Piekutowska, Donia H. Sheir, Lucas Leonardo da Silva, Camila da Silva Castro, Daniel Diego Costa Carvalho, Jadson Diogo Pereira Bezerra, Hosam A. Saad, Leonardo Luiz Borges, Solange Xavier-Santos

**Affiliations:** 1 Basic, Applied and Scientific Divulgation Mycolgy Laboratory (FungiLab), Central Campus, State University of Goiás, Anápolis 75132-903, GO, Brazil; igor.santos@aluno.ueg.br (I.R.d.S.); lucasleo.bio@gmail.com (L.L.d.S.); camila.castro@aluno.ueg.br (C.d.S.C.); leonardo.borges@ueg.br (L.L.B.); 2 Botany and Microbiology Department, Faculty of Science, Suez Canal University, Ismailia 41522, Egypt; 3 Botany and Microbiology Department, Faculty of Science, Damietta University, New Damietta 34511, Egypt; marwabotany@yahoo.com; 4 Department of Geoecology and Geoinformation, Institute of Biology and Earth Sciences, Pomeranian University in Słupsk, Partyzantów 27, 76-200 Słupsk, Poland; magdalena.piekutowska@apsl.edu.pl; 5 National Research Centre, Chemistry of Natural and Microbial Products Department, Pharmaceutical Industries Division, Giza 12622, Egypt; donia_sheir@yahoo.com; 6 Phytopathology Laboratory, Southeast Campus, State University of Goiás, Ipameri 75780-000, GO, Brazil; daniel.carvalho@ueg.br; 7 Mycology Sector, Department of Biosciences and Technology, Institute of Tropical Pathology and Public Health, Federal University of Goiás, Goiânia 74690-900, GO, Brazil; jadsonbezerra@ufg.br; 8 Department of Chemistry, College of Science, Taif University, P.O. Box 11099, Taif 21944, Saudi Arabia; h.saad@tu.edu.sa

**Keywords:** endophytic fungi, medicinal plants, bioprospecting, secondary metabolites, biotechnological potential, natural products

## Abstract

A multitude of plants from the Brazilian savanna are known for their medicinal properties. Many plants contain endophytic fungi, which lead to the production of bioactive compounds by both the fungi and their hosts. This study investigated the bioprospecting of endophytic fungi recovered from the leaves of *Palicourea rigida*, a native medicinal plant of the Brazilian savanna. Four fungal taxa (*Colletotrichum* sp. SXS649, *Pestalotiopsis* sp. SXS650, the order Botryosphaeriales SXS651, and *Diaporthe* sp. SXS652) were recovered. The phenolic, flavonoid, extracellular degrading enzymes (amylase, cellulase, protease, and tannase) and antioxidant activity of these taxa were determined. Evaluation of the antimicrobial activity showed that the Botryosphaeriales SXS651 extract displays a minimum inhibitory concentration (MIC) of 23.20 mg mL^−1^ against *Staphylococcus epidermidis* and *Pseudomonas aeruginosa*, and the *Diaporthe* sp. SXS652 extract exhibited an MIC of 27.00 mg mL^−1^ against *Escherichia coli*. The *Colletotrichum* sp. SXS649 isolate inhibited tumors in potato discs by 69% at a concentration of 9.70 mg mL^−1^. All isolates had potential bioremediation criteria against soil contaminated with soybean oil, as proved by a high percentage of germination of *Lactuca sativa* and a reduction in phytotoxicity. Furthermore, the taxa under investigation demonstrated antagonistic action to phytopathogenic fungi, namely, *Aspergillus niger*, *Inonotus rickii*, *Pestalotiopsis mangiferae*, and *Coniophora puteana*, with an inhibition range between 34.2% and 76.9%. The preliminary toxicity assessment showed that all isolates possessed an LC50 of less than 100 mg mL^−1^ to the microcrustacean *Artemia salina*. These results indicate that the endophytic fungi of the Brazilian savanna are promising candidates for biotechnological and industrial applications and, in agricultural applications, for the biological control of phytopathogenic fungi.

## 1. Introduction

The Brazilian savanna is the largest region of tropical savanna vegetation in the world and one of the main biodiversity hotspots, with many endemics and some threatened species [1,2]. A large portion of the savanna’s biodiversity lies in its medicinal plants, which are used in folk medicine or religious rituals [3]. Many of these plants have been processed by the pharmaceutical industry to obtain bioactive substances or used as herbal medicines [4]. Natural products containing secondary metabolites are important indicators of plant species bioactivity [5]. In addition to plants, endophytic microorganisms have also been explored to obtain these compounds [6]. Several plant tissues harbor endophytic microorganisms that protect against pests and pathogens, aiding plant growth, providing resistance to stress, and producing enzymes and other metabolites [7]. In turn, endophytic fungi receive nutrients and protection from the host [8]. The worldwide exploration of medicinal plants has led to a loss of diversity, and this reinforces the need for the investigation and discovery of different sources of bioactive compounds [9]. Fungi are valuable sources of natural products with diverse potentials [10,11].

Bioprospecting research in endophytes still needs much attention because few products from it are available in the pharmaceutical market [9,12]. The main biological activities of endophytic fungi are antimicrobial [13,14], antitumor [15,16], enzymatic [17], antioxidant [18,19,20], and insecticidal [20,21]. Several natural products of endophytic fungi isolated from medicinal plants in the Brazilian savanna are reported in the literature. The antimicrobial and anticancer activities of endophytes of *Stryphnodendron adstringens* (Mart.) [22] and *Baccharis trimera* (Less.) DC. Ref. [23] have been highlighted, as have the antioxidant and anticholinesterase activities of endophytic fungi of *Casearia sylvestris* Sw. [24]. 

Therefore, the importance of promoting studies to contribute to the knowledge and bioprospecting of endophytic fungi associated with medicinal species from the Brazilian savanna becomes evident, as previous studies, although scarce, present relevant results for biotechnology [22]. Microbial biotechnology has promoted new perspectives for applications of beneficial endophytic microbes for industry, agriculture and medicine, attracting the attention of the scientific community due to its adaptability in plants to extreme abiotic conditions [6].

In this perspective, *Palicourea rigida* Kunth (Rubiaceae) is a medicinal plant native to the savanna and used in folk medicine for the treatment of urinary disorders and disorders of the female reproductive system [25,26]. It is also used as a diuretic, hypotensive, healer, and treatment for stomach and kidney pain and for coughs [27]. However, the endophytic mycobiota of this species have not been reported yet, including their bioactive properties. This work aimed at promoting the bioprospecting of the biological activity of endophytic fungi isolated from *P. rigida* leaves in terms of their enzymatic, antioxidant, antimicrobial, antitumor, bioremediative, antagonistic, and toxicity potential.

## 2. Materials and Methods

### 2.1. Study Area and Sampling

**Figure 1:**
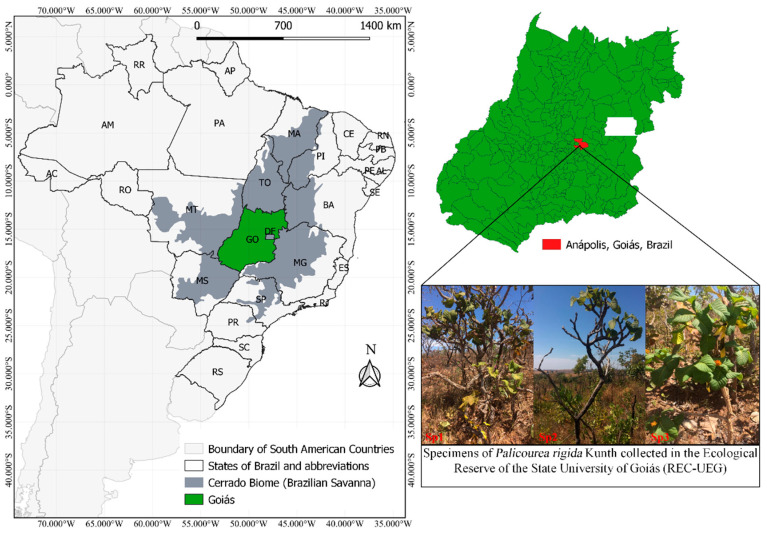
Geographic location of the UEG Ecological Reserve (REC-UEG), Anápolis, Goiás, Brazil and specimens of *Palicourea rigida* sampled.

*P. rigida* plants were collected from the UEG Ecological Reserve (REC-UEG), a fragment of the savanna *sensu stricto* located on the Central Campus of the State University of Goiás, Anápolis, Goiás (16°23′40″ S e 37°57′32″ W) (Figure 1). The area is characterized by the savanna *sensu stricto* phytophysiognomies, dry forest, and gallery forest [28]. The climate of the region is of the Cwb type (tropical of altitude, with dry winters and hot, humid summers) according to the Köppen classification [29].

Three healthy specimens of *P. rigida* from the savanna *sensu stricto* at the georeferenced points were selected: (1) 16°22′53″ S and 48°56′39″ W, (2) 16°22′43″ S and 48°56′34″ W, and (3) 16°22′14″ S and 48°56′30″ W. Samples were collected in sterilized polyethylene bags and transferred to the laboratory, where they were subsequently plated out. Samples were collected with the permission of the UEG Ecological Reserve (REC-UEG) for scientific purposes, and no endangered species were involved in the study. A herbarium sample was deposited in the Herbarium of the State University of Goiás (HUEG), under accession number HUEG 13846.

### 2.2. Isolation of Endophytic Mycobiota

Pieces of leaves were washed in running water to remove dust residues, immersed in 70% ethanol for 1 min, transferred to sodium hypochlorite solution (2% active chlorine) for 3 min, and again immersed in 70% ethanol for 30 s, followed by immersion twice in sterile distilled water. Fragments of approximately 3 to 5 mm^2^ were cut with the aid of scissors [30]. The fragments were transferred to potato‒dextrose‒agar (PDA) plates supplemented with the antibiotic chloramphenicol (100 µg mL^−1^) to inhibit bacterial growth. Plates were incubated at 27 ± 2 °C for up to 60 days and monitored weekly. Twelve endophytic fungi were isolated.

### 2.3. Phenotypic Identification

Identification of the recovered endophytic fungal isolates was conducted based on phenotypic means with the relevant identification keys [31,32,33,34]. For *Colletotrichum* recovered colonies were grown on PDA at 27 °C for morphological analysis. Taxonomic criteria include color of colony according to Raynor [35], texture and colony growth rate (diameter) were examined after 7 and 10 days. Conidia from the conidiomata in culture were mounted in lactic acid and the length and width measured for 50 randomly selected conidia with the range and mean calculated. For *Pestalotiopsis*, fungus was grown on PDA. Cultures were incubated at 27 °C in continuous light, and cultural morphology was examined after 7 days. Colony color was defined according to reported method [35]. Spore size was determined by measuring the length and width of 50 arbitrarily selected conidia from a conidial suspension of each colony that was prepared in sterile distilled water (SDW). The isolates were identified initially by comparing morphological and cultural characteristics (i.e., size of conidia, color and length of median cells, thickness and length of apical appendages, and length of basal appendage). 

Morphological characterization of *Diaporthe* was based on sporulating pycnidia from inoculated alfalfa stems placed on 1.5% water agar (WA). Whenever possible, 40–50 measurements were made of the structures mounted in 5% KOH. For determination of colony characteristics, PDA as inoculating medium was used at 27 °C in the dark. Phenotypic criteria of colony included diameters at intervals of 24 h for 1 week and used to calculate the growth rate of three replicates. After 1 week, colony size and color of the colonies and zonation were recorded [35].

Based on phenotypic identification, four taxa coded from SXS649to SXS652 showed vigorous growth and were selected for more bioprospecting of their biological activity. Cultures of these isolates were preserved according to [36] and deposited in the culture collection of the Basic, Applied Mycology, and Scientific Divulgation Laboratory at UEG (FungiLab) in Brazil and at Suez Canal University Fungarium (SCUF) (http://www.wfcc.info/ccinfo/index.php/collection/by_id/1180/, accessed on 20 August 2021) in Egypt under accession numbers SCFU0001891 to SCUF0001894. Representative endophytic isolates were also deposited in the working collection of Jadson D. P. Bezerra (Fungi Culture Collection, Federal University of Goiás [FCCUFG] at the Institute of Tropical Pathology and Public Health, Federal University of Goiás, Goiânia, Brazil.

### 2.4. Extraction of Active Metabolites 

The taxa under investigation were grown on PDA plates for 7 days and transferred to Erlenmeyer flasks (500 mL) containing 300 mL of Czapek–Dox broth (NaNO_3_, 3 g; K_2_HPO_3_, 1 g; MgSO_4_·7H_2_O, 0.5 g; KCl, 0.5 g; FeSO_4_, 0.01 g; sucrose, 30 g; distilled water, 1000 mL) according to the method of Bagheri et al. [37]. Cultures were incubated for 30 days at 27 °C on a rotary shaker at 150 rpm. The fermentation broth of each taxon was filtered; the filtrate so obtained was stored at 10 °C and used as crude fungal extract for different assay procedure. 

### 2.5. Determination of Solids Content

To determine the solids content of each fungal extract, a 1-mL aliquot was placed on a balance with a halogen heating element (Ohaus-MB35) and subjected to 105 °C for 5 to 10 min. The result was expressed as the average of three determinations, in percentages, thus obtaining the solids content by difference (% volatile content − 100 = % solids content). The total concentrations of the fungal extract are shown in Table 1, as well as the four standardized concentrations used for the evaluation of the antioxidant, antimicrobial, antitumor, and toxicity activities.

### 2.6. Qualitative and Quantitative Screening of Secondary Metabolites

Qualitative screening of fungal extracts for the presence of saponins, steroids, alkaloids, phenols, and flavonoids was carried out in triplicate, according to previously reported methods of [38,39,40].

Total phenolic content: For the quantitative determination of the total phenolic content, the work of [41] was consulted and adopted. Aliquots of 1 mL of each fungal extract were placed in test tubes, 0.6 mg mL^−1^ of gallic acid solution 10% was added, and the mixture was subsequently transferred to volumetric flasks containing 2 mL of distilled water. An amount of 100 µL of gallic acid solution was added to the test tubes, as well as 2 mL of Folin‒Ciocalteu reagent 3%. After 5 min, 2 mL of sodium carbonate (Na_2_CO_3_) solution 15% (*m*/*v*) was added, and the volume was brought up to 900 µL with distilled water. After 120 min in the absence of light, the absorbance was measured at 760 nm by a digital spectrophotometer (Mod. IL-592-Kazuaki), using gallic acid solution 10% as the standard, at concentrations of 200, 400, 600, 800, and 1000 µL for construction of the curve. Using the equation for the straight line obtained in the curve of the pattern graph, the total phenol content was calculated, with the results expressed in milligrams of gallic acid in 1 mL of fungal extract.

Total flavonoid content: For determination of the total flavonoid content, the formula of [42] was applied. Rutin 95% (3-*O*-rutinoside-quercetin) 0.1 mg mL^−1^ was used as a standard solution. Aliquots of 1 mL of fungal extract were added to 100 mL of 0.02-M acetic acid methanol solution 99%. After the solution had rested for 40 min in the absence of light, the absorbance was measured at 316 nm by a digital spectrophotometer (Mod. IL-592-Kazuaki). The total flavonoid content was determined using a standard rutin curve at concentrations of 100, 200, 300, 400, and 500 µL, and the volume was brought up to 2 mL with a methanol solution 25 mg mL^−1^. Using the equation of the straight line obtained in the curve of the standard graph, the total flavonoid content was calculated, with the results expressed in milligrams of rutin in 1 mL of fungal extract.

HPLC analysis: The extracts were analyzed by high-performance liquid chromatography (HPLC) to separate the components from the samples. Analyses were performed with a Waters model HPLC Alliance chromatograph with an e2695 separation module, 2998 diode array detector (DAD), and Empower 2.0 data-processing system. Chromatographic separations were carried out on a Zorbax Eclipse XDB-C18 reversed-phase column (250 × 4.6 mm^2^, 5 µm), using 0.05% formic acid in HPLC-grade acetonitrile (pH = 3.45) (solvent A) as a mobile phase and 0.05% formic acid in ultrapure water (Mili-Q) (pH = 3.15) (solvent B) at a flow of 1 mL min. The following mobile phase gradient was applied: 0% to 5% A (0 to 5 min); 5% to 10% A (5 to 15 min); 10% to 15% A (15 to 25 min), 15% to 20% A (25 to 35 min), and 15 min isocratic 20% A (35 to 50 min). The injection volume was 10 μL at 35 °C. The mobile phase was previously filtered through a 0.45-µm Millex^®^ polyvinylidene fluoride (PVDF) filter (Merck KGaA, Darmstadt, Germany ) and degassed in an ultrasonic bath (USC 1800A, 40 kHz, Unique Group, São Paulo, Brazil).Thus, 1 mL of each fungal extract was diluted in 2 mL of HPLC-grade methanol and the standards (Sigma Aldrich, Sigma-Aldrich Brasil Ltda. Av. Queirós Filho, 1319 - Vila Humaita, Santo André, Brazil ) were prepared at a concentration of 0.25 mg mL^−1^ in HPLC-grade methanol. The qualitative presence of caffeic acid, chlorogenic acid, ellagic acid, gallic acid, *p*-coumaric acid, caffeine, catechin, epicatechin and naringenin in each fungal extract was evaluated. The wavelengths used for detection were 254, 327, and 366 nm. The compounds in the extract were identified by comparing the retention times (Tr) and the ultraviolet (UV) absorption spectrum (190 to 400 nm) of the peaks obtained. The solutions were previously filtered through a 0.45-μm Millex^®^ membrane.

### 2.7. Extracellular Degradative Enzymes

The activity of the amylase, cellulase, protease, lipase, pectinase, and tannase enzymes was assayed by techniques adapted from the work of [43,44]. Taxa were cultivated on PDA plates for 5 days, in a bio-oxygen demand (BOD0 incubator), at 27 °C. During this period, a culture disc of 6 mm^2^ in diameter was inoculated in the center of the Petri dish containing a solid basal minimal medium (NaNO_3_, 6 g; KH_2_PO_4_, 1.5 g; MgSO_4_·7H_2_O, 0.5 g; FeSO_4_, 0.02 g; ZnSO_4_, 0.02 g; agar, 20 g; distilled water, 1000 mL), with each of the respective carbon sources, that is, corn starch (5 g) for detection of amylases; 10% carboxymethylcellulose (5 g) for celluloses; skimmed milk (20 g) for proteases; Tween 80 (5 mL) for lipases; pectin (5 g) for pectinases; and tannic acid (5 g) for tannases. The plates were incubated at 25 °C for 5 days. This allowed time for the presence or absence of halo formation around the colony to indicate the enzyme production capacity. To reveal the halo of amylases, lugol solution was used; for cellulases, a 0.1% congo red solution was used and then the plates were washed with NaCl. For proteases, 1% methylene blue solution was used; for pectinases, lugol solution was added and washed with water; for lipases, the presence of crystals was verified; and for tannases, a blackened halo was used. The determination of activity was expressed by the enzymatic index (IE) obtained with the formula IE = diameter of the halo/diameter of the colony, according to [45]. Isolates with an IE of 2.00 or greater were those with the highest extracellular enzymatic activity. The tests were carried out in triplicate.

### 2.8. Antioxidant Activity

To verify the antioxidant activity of the fungal extracts, two methods were selected. The first was based on free radical scavenging by using 2,2-diphenyl-1-picryl-hydrazyl-hydrate (DPPH) and the second on the ferric-reducing ability of plasma (FRAP). The DPPH activity was carried out according to a technique adopted from [46,47]. Each fungal extracts (Table 1) were diluted in 2 mL of DPPH and 10% methanol (MeOH) solution (25 μg mL^−1^) to obtaining the values of the solutions 0, 200, 400, 600, 800, and 1000 µL mL^−1^. The mixture was incubated for 30 min at 37 °C in the absence of light, and the absorbance was measured at 515 nm by a digital spectrophotometer (Mod. IL-592-Kazuaki). For comparison with the antioxidant activity of the fungal extract, a standard curve was prepared with a synthetic antioxidant, butylhydroxytoluene (BHT) (25 μg·mL^−1^). The negative control was formed by replacing the sample volume with an equal volume of MeOH, and for the blank, only MeOH was used. The concentration capable of inhibiting 50% of the DPPH radical activity (IC50) was calculated by linear regression and expressed as the mean ± standard deviation, where the x-axis represented the concentration (mg mL^−1^) and the y-axis represented the initial absorbance of the control divided by 2. The percentage of antioxidant activity was calculated using the equation AA% = [(Ac − A) ÷ Ac] × 100, where A is the absorbance value of the sample and Ac is the absorbance value of the control solution.

The FRAP method evaluates the antioxidant power of iron reduction; it exclusively involves electron transfer reactions in which the reduction of the TPTZ complex (2,4,6-tri(2-pyridyl)-1,3,5-triazine) occurs with Fe^3+^ to Fe^2+^ [48]. Each fungal extracts (Table 1) were diluted in 1 mL distilled water to obtain concentrations of 0, 20, 40, 60, 80, and 100 mL–1μL. As a standard, a 2.0-mM ferrous sulfate solution was used, from which the analytical curve points were prepared at different concentrations 100, 200, 300 e 400 μM). The FRAP reagent was prepared at the time of analysis by mixing 50 mL of potassium acetate buffer (0.3 M, pH 3.6), 5 mL of TPTZ solution (10 mM TPTZ in 40 mM HCl), and 5 mL of ferric chloride (20 mM) in an aqueous solution. The negative control was formed by replacing the sample volume with an equal volume of distilled water. For the white, only distilled water was used instead of the fungal extract. In a 96-well microplate were added 100 μL of the FRAP reagent to each well. The plate was incubated at 37 °C for 8 min and then submitted to an absorbance reading at 595 nm in a microplate spectrophotometer (SpectraMax-M3, Molecular Devices, LLC., 3860 N First Street San Jose, CA, USA). The antioxidant activity was expressed according to the absorbance equivalent to 1000 μM of the ferrous sulfate standard, calculated in the equation y = ax + b, where y is the absorbance corresponding to 1000 μM of ferrous sulfate and ax is the sample dilution (mg mL^−1^) equivalent to 1000 μM of ferrous sulphate.

### 2.9. Antimicrobial Activity

For the evaluation of the antimicrobial activity, the minimum inhibitory concentration (MIC) was determined through microdilution tests in extract according to the Clinical and Laboratory Standard Institute [49], with modifications. Bacterial strains *Staphylococcus aureus* ATCC25923, *Staphylococcus epidermidis* ATCC12228, *Escherichia coli* ATCC25312, *Pseudomonas aeruginosa* ATCC27853, and *Klebsiella pneumoniae* ATCC700603 and the yeast strains *Candida albicans* ATCC10231 and *Candida tropicalis* ATCC13860 were used. Bacterial taxa were inoculated in Mueller Hinton (MH) agar and incubated at 35 °C for 24 h, and yeast strains were inoculated in Sabouraud agar and incubated at 35 °C for 24 h.

Bacterial and yeast strains were inoculated singly in a tube containing 4.5 mL of 0.9% saline solution until obtaining a turbidity corresponding to 0.5 on the McFarland scale. The solution was diluted 1:100 in saline solution, reaching a concentration of 1106 CFU mL^−1^ and then inserted into MH broth and Sabouraud broth. The procedure was performed 20 min before inoculation into the microplate wells. The fungal extracts were solubilized in MH broth and Sabouraud broth, with serial dilutions in a ratio of 1:1000.

The dilutions of the antibiotic chloramphenicol and the antimycotic miconazole nitrate were prepared according to CLSI recommendations as controls [49]. After preparation of the dilutions, the fungal extracts (Table 1) were diluted in 100 μL of MH broth and Sabouraud broth in the microplate wells. As a viability control, 100 μL of MH broth and Sabouraud broth were pipetted with the inoculum. As a control for the extracts, 200 μL of MH and Sabouraud broth without inoculum and 200 μL of each fungal extract were pipetted. Then, 100 μL of each inoculum was deposited in all wells of the plate, except for the wells that were used to control the sterility of the medium and samples. The microplates were sealed and incubated at 35 °C for 24 h. After the incubation period, 25 μL of 0.1% sodium resazurin was added to all wells. Then the plates were again incubated for 30 min. The absorbance reading was performed by comparing the turbidity of the samples in the wells before adding 0.1% sodium resazurin and after adding 0.1% sodium resazurin, considering that the increase in turbidity or opacity in the medium was considered indicative of the growth of microorganisms. This followed the methodology of [50], in which the permanence of the blue color means inhibition of the growth of microorganisms and the pinkish red color means metabolic activity due to the growth of microorganisms. The MIC is defined as the lowest concentration of the compound in mg mL^−1^ capable of totally inhibiting bacterial growth visible to the naked eye.

### 2.10. Antitumor Activity

To assess the antitumor activity, *Rhizobium radiobacter* ATCC4720 was cultivated in tryptone soy agar (TSA) medium for 24 h and then a bacterial suspension was prepared at a concentration of 1108 CFU.mL^−1^. It was determined through comparison with the McFarland scale and the dilution of 1:10 in nutrient broth, obtaining a suspension of 1.106 CFU.mL^−1^, which was incubated for 48 h at 36 °C for subsequent incubation of the potato discs [51].

Fresh and peeled medium-sized potatoes (*Solanum tuberosum* L.) had their surfaces disinfected by immersion in 0.1% NaClO for 30 min. Then, potato discs (5 × 8 mm^2^) were extracted from the core region, using a metal awl. The potato discs were placed in Petri dishes containing bacteriological agar, and each dish received three discs for each dilution of fungal extract. Each inoculation mixture was prepared with 1900 µL of bacterial culture suspension, 0.5 mL of distilled water and the fungal extracts (Table 1) in 100 µL of TSA, thus obtaining the final concentrations of 1000, 500 and 250 µL of this solution for pipetting onto potato discs. Then, 100 μL of each inoculation mixture was inoculated into each potato disc, and the discs were incubated for 20 days at 25 °C. The technique’s control discs were made in the same way, except that the fungal extracts were removed. Vincristine (100 µg mL^−1^) was used as a positive control due to its significant effect on tumor inhibition [52]. During this period, the reading was performed 30 min after the addition of lugol solution (5% I2 + 10% KI) and tumor counts were performed and compared with the control technique. The results were presented in percentages using the formula 100 – mean tumors found in the sample/mean tumors found in the growth control ×100. Significant activity was indicated by 20% or more inhibition.

### 2.11. Bioremediative Activity

To evaluate the potential of fungal extracts for bioremediation of soil contaminated with soybean oil, a soil phytotoxicity reduction test was carried out using lettuce seeds (*Lactuca sativa* L.) as modified from [53]. Soil samples were collected from the same locations where the host plants *P. rigida* were collected. The soil retention capacity was determined following ISO 17126:2005. By using 300-mL plastic cups perforated in the base, 100 g of soil sieved in a stainless-steel sieve with 16 openings (1.18 mm^2^) was weighed and placed in a container with water until saturation for 15 h to drain the water surplus. During this period, the cups were weighed to obtain the wet soil mass, using the following formula: C = mw – md, where C is the retention capacity (100%), mw is the wet soil mass in grams, and md is the dry soil mass in grams. Thus, the maximum water retention capacity in 100 g of soil was found. The concentration of soybean oil considered toxic to *L. sativa* seeds is 10% (10 mL).

Using 300-mL plastic cups perforated in the base, 100 g of soil was weighed in triplicates. For control 1, soil, water, and 10 lettuce seeds were placed in each cup; for control 2, soil, water, 10% soybean oil (10 mL), and 10 lettuce seeds were placed in each cup; and for the soil being treated, 10% soybean oil (10 mL) and the value of C (retention capacity, as obtained previously) of the fungal extract *n* = 32 mL, were placed in each cup. The preparations were incubated in a germination oven for 144 h at 25 °C. Afterward, the percentage of germination and length of roots and hypocotyls were verified and were compared with the controls.

### 2.12. Antagonistic Activity

The antagonism evaluation was carried out by dual cultures in plates [54]. Direct inoculation of each recovered endophytic fungus was made against the phytopathogenic fungi isolates, namely, *Aspergillus niger* SXS635 (black mold fungus in fruits), *Inonotus rickii* SXS37 (wood rot fungus), *Pestalotiopsis mangiferae* SXS647 (leaf spot fungus of *Mangifera indica*), and *Coniophora puteana* SXS623 (wood rot fungus). Each Petri dish containing PDA medium received two paired 6-mm^2^ discs equidistantly matched, 1 cm^2^ from the edge, one disc from the edge of the endophytic fungus colony and the other disc from the edge of the phytopathogenic fungus colony. Then, the plates were incubated at 27 °C for 14 days. As a control technique (control), the phytopathogenic fungus was inoculated without the presence of endophytic fungi in one pole of the plate.

The competitive interactions between endophytic fungi and phytopathogens were evaluated following the scale of [55]. Three possible types of interactions were expected: A, “deadlock” with mycelial contact; B, distance “deadlock”; and C, endophytic growth on the phytopathogen without initial deadlock, being divided into four subcategories (CA1, CA2, CB1, and CB2). CA1 and CA2 were the partial and complete growth of the endophytic fungus on the phytopathogen after initial deadlock with mycelial contact, and CB1 and CB2 were the partial and complete growth of the endophyte on the phytopathogen after the initial distance deadlock. In addition, to verify the percentage of growth inhibition of the phytopathogen, the area of these compared to the control area was measured, using the formula Percentage of inhibition of phytopathogen growth (PI%) = (Dc − Dt/Dc) × 100, where Dc is the average diameter of the colony of the phytopathogen of the control plates (without antagonist) and Dt is the average diameter of the colony of the phytopathogen against the antagonist (isolated endophytic) [56].

### 2.13. Toxicity

The toxicity of the fungal extracts was evaluated against *Artemia salina* Leach. by a modified technique of [57]. Synthetic sea water was prepared by dissolving sea salt (36 g L^−1^) in distilled water, with the addition of a yeast extract (6 mg L^−1^). The solution was autoclaved at 120 °C for 15 min. An aliquot of 250 mg of *A. salina* cysts was incubated for 37 h in 500 mL of synthetic marine water, under natural lighting and constant oxygenation at 25 °C. After the incubation period, with the hatching of the cysts and the release of the nauplii, they were attracted by a light source, pipetted, and transferred to 96-well polystyrene microplates. Inoculums were standardized on 10 ± 1 nauplii for each well containing 100 µL. The fungal extracts (Table 1) were diluted in 100 µL of synthetic seawater, then a further 100 µL of synthetic seawater with nauplii was added. 

An amount of 10 µL of synthetic marine water added to 190 µL of sterilized distilled water was used as a control. For lethality, potassium dichromate (K_2_Cr_2_O_7_) at concentrations of 100, 50, 25, 12.5, and 6.25 μg was used [58]. The determination of the LC50 was performed from the count of dead and alive nauplii, after 24 h of exposure to the extracts, using a stereoscopic optical microscope (Probit dose‒response methodology through Statplus version 5.7.8, 2014 professional [Analyst Soft]). Linear regression analysis was obtained from the relationship between the percentage of dead nauplii and the concentration of fungal extracts. The level of toxicity was determined according to [59] and [59]: LC50 < 100 mg mL^−1^ strong, 100 to 500 mg mL^−1^ moderate, 500 to 1000 mg mL^−1^ low, >1000 mg mL^−1^ nontoxic.

### 2.14. Statistical Analysis

Results were shown as the mean ± standard deviation. The analysis of variance (ANOVA) followed by the Tukey test was used to measure the significance level at 5% in the means of the assessments of bioremediation and antagonism activities using BioEstat 5.3 software.

## 3. Results

The endophytic fungi recovered from *P. rigida* were identified phenotypically as *Colletotrichum* sp. SXS649- SCFU0001891 (Glomerellaceae: Glomerellales: Sordariomycetes), *Pestalotiopsis* sp. SXS650- SCFU0001892 (Sporocadaceae: Xylariales: Sordariomycetes), Botryosphaeriales SXS651- SCFU0001893 (Dothideomycetes), and *Diaporthe* sp. SXS652- SCFU0001894 (Diaporthaceae: Diaporthales: Sordariomycetes), all belonging to the phylum Ascomycota. Taxonomically they were belonged to 1 class, 4 orders and three families. 

**Figure 2:**
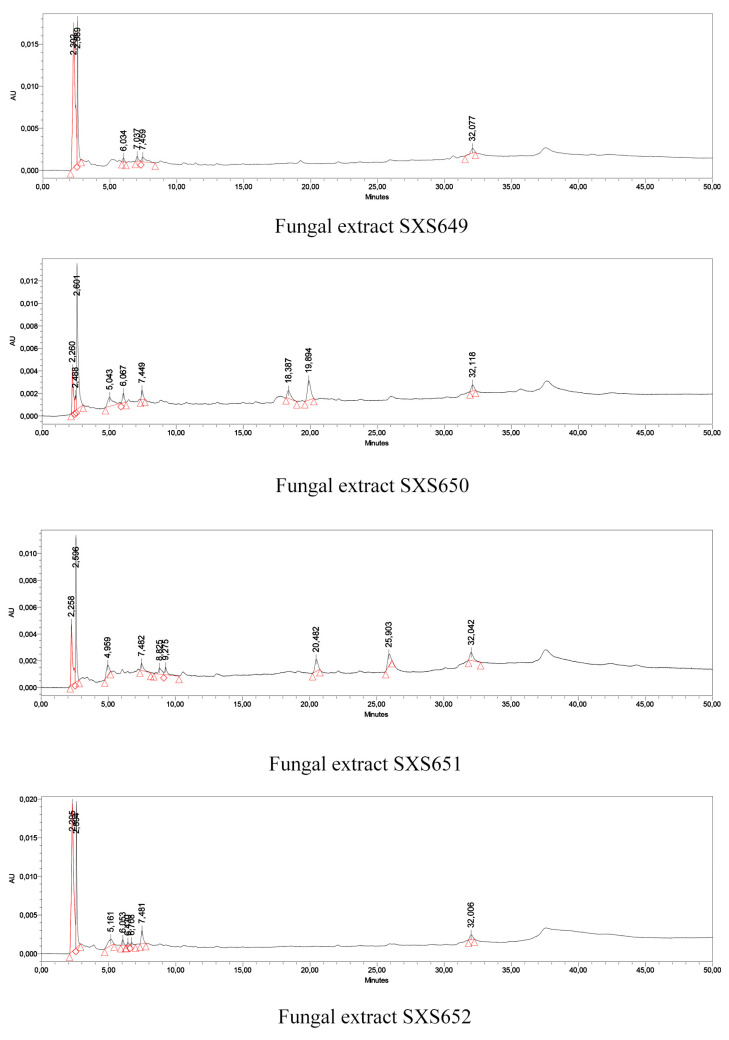
HPLC chromatograms of extracts of the endophytic fungi *Colletotrichum* sp. SXS649, *Pestalotiopsis* sp. SXS650, Botryosphaeriales SXS651, and *Diaporthe* sp. SXS652 isolates from *P. rigida* Kunth. Values correspond to the average of 3 repetitions.

Qualitative screening for secondary metabolites confirmed the presence of phenols and flavonoids in the extracts of all studied isolates, but no detectable content of saponins, steroids and alkaloids. The quantification of the total phenolic compounds and flavonoids showed a content of 210 to 890 mg of a total phenolic equivalent of gallic acid per milliliter of extract and 84 to 374 of a total flavonoid equivalent of rutin per milliliter of extract. The *Colletotrichum* sp. SXS649 had the highest amount of both metabolites (Table 2). The analysis of the resulting HPLC-DAD chromatograms suggested the presence of compounds in the four fungal extracts (Figure 2); however, none of these compounds were similar to the analyzed patterns caffeic acid, chlorogenic acid, ellagic acid, gallic acid, *p*-coumaric acid, caffeine, catechin, epicatechin and naringenin. Further research is needed to identify the compounds. 

**Figure 3:**
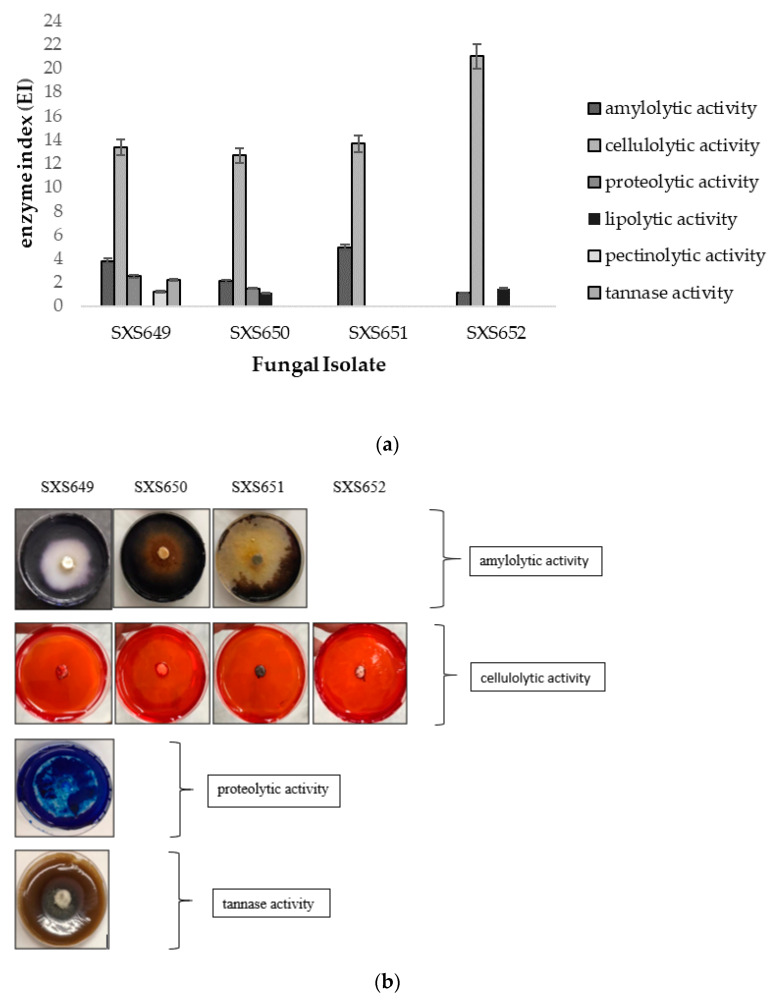
Enzymatic profile of the endophytic fungi *Colletotrichum* sp. SXS649, *Pestalotiopsis* sp. SXS650, Botryosphaeriales SXS651, and *Diaporthe* sp. SXS652 isolates from *P. rigida* Kunth. (**a**) Enzyme index (IE). Values correspond to the average of 3 repetitions: (**b**) Cultures in specific media showing the substrate degradation halo as indicative of enzymatic activity.

Degradative enzymes’ activity showed that all isolates had high cellulolytic activity, with an IE between 12.66 and 21.00. *Colletotrichum* sp. SXS649, *Pestalotiopsis* sp. SXS650, and Botryosphaeriales SXS651 showed amylolytic activity, with an IE of 3.80, 2.10, and 4.90, respectively. *Colletotrichum* sp. SXS649 showed proteolytic activity with an IE of 2.53 and tannase activity with an IE of 2.21. No isolate showed lipolytic or pectinolytic activity, with an IE of below 2.00 (Figure 3).

Fungal extracts showed a potential antioxidant activity in the DPPH assay, although high concentrations were needed to reach the same capture percentage as the positive control with butylated hydroxytoluene (BHT). The IC50 detected among the isolates is shown in Table 3. Only 0.0118 mg mL^−1^ of BHT was needed to achieve results similar to those of the extracts. For the percentage of antioxidant activity through free radical capture (AA%) to reach an average of 65.48% (the greatest potential), it was necessary to have a concentration of 11.60 mg mL^−1^ of the extract of the isolate Botryosphaeriales SXS651 e 0.015 mg mL^−1^ of BHT. For the FRAP assay, extracts from *Colletotrichum* sp. SXS649, *Pestalotiopsis* sp. SXS650, and *Diaporthe* sp. SXS652 presented results corresponding to the sample dilution equivalent to 1000 μM of ferrous sulfate. *Pestalotiopsis* sp. SXS650 and *Diaporthe* sp. SXS652 showed antioxidant activity at moderate levels with the DPPH and FRAP; *Colletotrichum* sp. SXS649 showed lower levels of antioxidant activity with the DPPH and FRAP methods; and Botryosphaeriales SXS651 showed lower levels of antioxidant activity with the DPPH method.

**Figure 4:**
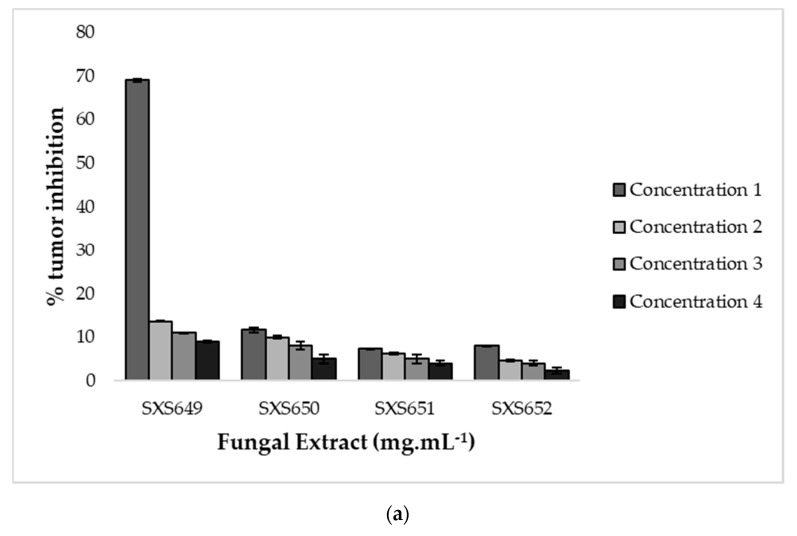

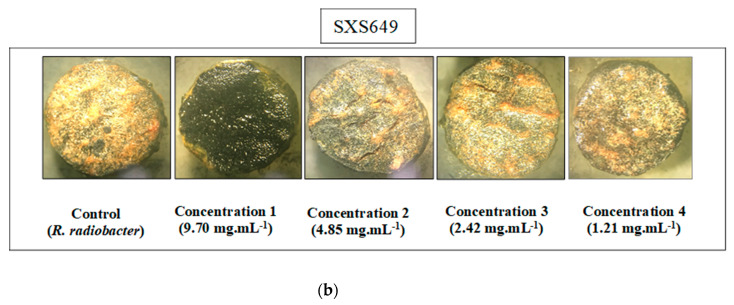
Antitumor activity in potato discs of extracts of the endophytic fungi *Colletotrichum* sp. SXS649, *Pestalotiopsis* sp. SXS650, *Botryosphaeriales* SXS651, and *Diaporthe* sp. SXS652 isolates from *P. rigida* Kunth. (**a**) Inhibition rate against the concentration of fungal extracts. Values correspond to the average of 3 repetitions; and (**b**) Potato disc tumors treated with *Colletotrichum* sp. SXS649.

In verifying the antimicrobial activity, the extract of Botryosphaeriales SXS651 was able to inhibit the bacteria *S. epidermidis* and *P. aeruginosa* at a concentration of 23.20 mg mL^−1^, and the extract of *Diaporthe* sp. SXS652 showed inhibition of *E. coli* at a concentration 27.00 mg mL^−1^. In verifying the antitumor activity, only *Colletotrichum* sp. SXS649 showed tumor inhibition (69%) at a concentration of 9.70 mg mL^−1^. The other tested concentrations of this isolate showed inhibition of less than 20% (Figure 4). The extracts from the other fungal isolates did not show antitumor activity and did not inhibit tumor formation by more than 20%.

In verifying the bioremediation capacity of soil contaminated with soybean oil, the maximum water retention capacity in 100 g of soil was 32 mL. Therefore, this was the volume of water or fungal extract added to the soil for the tests. When the soil was contaminated with 10% soybean oil there was a reduction in the number of germinated seeds (Table 4). In the soil treated with fungal extracts there was an increase in seed germination, with emphasis on treatment with *Colletotrichum* sp. SXS649, where a germination rate of 72.5% (32.5% reduction in phytotoxicity) was verified. This was followed by treatment with *Pestalotiopsis* sp. SXS650, with a 67.5% germination rate (27.5% phytotoxicity reduction); Botryosphaeriales SXS651, with a 65% germination rate (25% phytotoxicity reduction), and *Diaporthe* sp. SXS652, with a 60% germination rate (20% reduction in phytotoxicity). This same phytotoxicity reducing effect was observed on the root and hypocotyl length.

**Figure 5:**
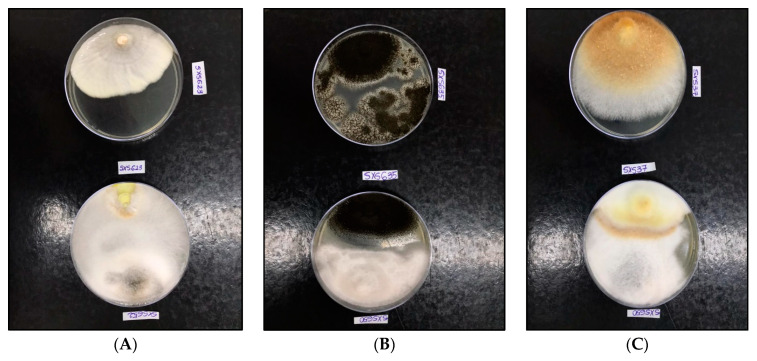
Paired culture test of endophytic fungi isolated from *P. rigida* Kunth against phytopathogenic fungi at 14 days of incubation. The plates arranged in the upper row correspond to the control and in the lower row to the test. (**A**): Above *C. puteana* SXS623 and below the same fungus × *Diaporthe* sp. SXS652 (PI 76.93%). (**B**): Above *A. niger* SXS635 and below the same fungus × *Pestalotiopsis* sp. SXS650 (PI 59.25%). (**C**): Above *I. rickii* SXS37 and below the same fungus × *Pestalotiopsis* sp. SXS650 (PI 57.14%).

All isolates showed antagonistic activity against tested phytopathogenic fungi, with a percentage of inhibition (PI%) of between 34.17% and 76.93% (Table 5). All of the endophytes showed an inhibition percentage of greater than 50% for at least one of the phytopathogenic fungi tested. The three highest percentages of inhibition were against *C. puteana* (76.93%), *A. niger* (59.25%), and *I. rickii* (57.14%) (Figure 5). In the verification of toxicity, all of the fungal extracts showed high lethality for the microcrustacean *A. salina* (LC50 < 100 mg mL^−1^) (Table 6).

## 4. Discussion

The taxon *Colletotrichum* has been reported by several investigators as an endophyte in almost all major groups of angiosperms [60,61,62,63], and it is still characterized as an important causative pathogen of anthracnose, preharvest and postharvest fruit rots, flower dampening, and seedling burning diseases in various plant species, especially cereals, throughout the world [64,65]. Thus, *Colletotrichum species* are among the most common endophytes and pathogens in leaf structures of terrestrial plants, with a record of approximately 2200 plant species [66]. In medicinal species from the Brazilian savanna, *Colletotrichum* has already been endophytically isolated from *Schinus terebinthifolius* [67], *Hancornia speciosa* [68], *C. sylvestris* [24], and *S. adstringens* [22]. This is the first record of it in *P. rigida*.

The literature has demonstrated the presence of phenolic compounds and flavonoids in *P. rigida*. Ref. [26] verified significant levels of these phenolic compounds in a polar fraction (933.25 µg·mL^−1^) of the leaves, and also the isolation of the flavonoids quercetin 3-*O*-β-d-glycoside, quercetin 3-*O*-sophoroside, and isorhamnetin 3-glycoside. In a phytochemical study of ethanol extracts from the leaves of this plant, Ref. [69] detected the presence of flavonoids at concentrations higher than standard rutin (440.5 and 365.5 mg mL^−1^, respectively). Ref. [70] reported the presence of phenolic compounds in *Palicourea* and other species of the Rubiaceae family. The detection of these metabolites in endophytic fungi isolated from it confirms the similarity in production, in which host and endophytes have metabolic exchanges [71].

Notarte et al. detected the presence of phenols and flavonoids, as well as sterols and terpenoids, in *Colletotrichum* extracts isolated from the medicinal plant *Uvaria grandiflora* [72]. These compounds showed antioxidant activity with ferric reducing power (4.41 mg mL^−1^) and superoxide scavenging activity (0.78 mg mL^−1^), in addition to high toxicity against the microcrustacean *A. salina* with a lethal dose of less than 0.40 mg mL^−1^. Ref. [73] isolated three *Colletotrichum* species (*Colletotrichum gloeosporioides*, *Colletotrichum tropicale*, and *Colletotrichum siamense*) from the medicinal plant *Justicia gendarussa*. They showed lower and moderate antioxidant activity with the DPPH elimination method and strong cytotoxic activity and lethality to *A. salina*, suggesting that the extracts have bioactive metabolites, which have been identified as flavonoids, coumarins, terpenoids, and steroids. In the Brazilian savanna, secondary metabolites of *Colletotrichum crassipes* isolated from the leaves of the medicinal plant *C. sylvestris* exhibited potent antifungal activity against the phytopathogenic fungi *Cladosporium cladosporioides* and *Cladosporium sphaerospermum* [24].

The genus *Pestalotiopsis* has also been reported as endophytic of the medicinal plant *Baccharis trimera* from the savanna, demonstrating antifungal activity against *Paracoccidioides brasiliensis* [23]. Fungi of the order Botryosphaeriales have also been reported in the global literature as endophytic, living in plant tissues for long periods. However, other species of the order are phytopathogens and some cause opportunistic infections in humans [74,75,76] isolated 52 endophytes of the order Botryosphaeriales from *Distylium chinense*, which showed significant antioxidant activity and antimicrobial activity against at least one of the microorganisms evaluated by the authors. *Diaporthe* sp., isolated from the leaves of the medicinal plants *Vernonia polyanthes* and *Anacardium othonianum* from the savanna, demonstrated antimicrobial activity against *Leishmania amazonensis* [77] and were able to solubilize phosphate [78]. Gomes et al. reported a large number of *Diaporthe* sp. obtained from medicinal plants in Brazil, suggesting that these plants are repositories for this genus [79]. Noriler et al. found antibacterial activity in isolates of *Diaporthe* sp. obtained from *S. adstringens* [80].

In verifying the enzymatic activity of the isolates obtained from *P. rigida*, *Colletotrichum* sp. showed the highest enzyme production of amylase, cellulase, protease, and tannase, followed by *Pestalotiopsis* sp. and the order Botryosphaeriales, with the production of amylase and cellulase. These enzymes could be used in industry. In verifying the antioxidant activity, all isolates showed lower and moderate levels, especially *Pestalotiopsis* sp., which had an IC50 of 47.06 mg mL^−1^ with the FRAP method. Two isolates, Botryosphaeriales and *Diaporthe* sp., showed antimicrobial activity against *S. epidermidis*, *P. aeruginosa*, and *E. coli*, making them candidates for therapeutic use. In the verification of antitumor activity in potato discs, *Colletotrichum* sp. showed inhibition of tumors in 69%, a result that confirmed that this isolate exhibit an antitumor bioactivity. Ref. [81] also verified that *Colletotrichum* sp., obtained from the leaves *of Artocarpus heterophyllus*, can be promising antitumor agents.

In addition, all isolates showed bioremediative activity in soil contaminated with soybean oil, with rates above 20% in the reduction in phytotoxicity. There was an increase in rates of germination and the length of roots and hypocotyls of *L. sativa*; the enzymes produced by the microorganisms underwent activation of the catalytic site in the presence of hydrophobic substrates, aiding in this activity [82]. Bibi et al. demonstrated that endophytic fungi obtained from healthy plants near the university campus in Mardan, Pakistan, promoted the bioremediation of chromium-contaminated soil by assay of *L. sativa* [53]. Russell et al. demonstrated the bioremediative potential of endophytic isolates of the *Pestalotiopsis genus*, which showed the ability to degrade the synthetic polyester polyurethane polymer [83].

Antagonistic tests against *A. niger*, *I. rickii*, *P. mangiferae*, and *C. puteana* had the following results: in 55% of the tests, the endophytic fungi blocked mycelial contact with the phytopathogenic fungi, in 25% they presented distance blocking to the phytopathogenic fungi, and in 20% they showed mycelial contact and partial growth on the phytopathogenic fungi. This interaction between endophyte and phytopathogen was marked by accelerated mycelial growth. Similar to the *Colletotrichum* sp. of our study, Ref. [84] found that *Xylaria regalis*, isolated from *Thuja plicata*, inhibited the phytopathogenic fungus *A. niger*. The brown rot fungus *C. puteana*, which causes a wood mass loss of up to 70% [85], was significantly inhibited by *Diaporthe* sp., indicating that this endophyte can be an efficient antagonist of *C. puteana* in the field. The verification of the toxicity of the isolates indicated a high lethality to the microcrustacean *A. salina*, with an LC50 of less than 100 mg mL^−1^. This indicates that caution is needed in using it for biotechnological procedures and that new tests verifying the toxic levels are necessary. However, its environmental uses are quite favorable, either for soil bioremediation or for the biological control of phytopathogens.

## 5. Conclusions

Fungi isolated from *P. rigida* (*Colletotrichum* sp. SXS649, *Pestalotiopsis* sp. SXS650, Botryosphaeriales SXS651 and *Diaporthe* sp. SXS652, native to the Brazilian savanna, proved to be producers of phenols and flavonoids in addition to amylases, cellulases, proteases, and tannases. Furthermore, they presented antioxidant, antimicrobial activity against *S. epidermidis*, *P. aeruginosa* and *E. coli*, 69% antitumor activity in potatoes, bioremediative activity in soil with soybean oil, and antagonistic action toward the phytopathogenic fungi *A. niger*, *I. rickii*, *P. mangiferae*, *and C. puteana*. All of the fungal extracts promote a high lethality rate in *A. salina*. These data indicate that these fungi present biological activity of biotechnological interest, encouraging their applicability in soil bioremediation processes, in the control of phytopathogens, however, further investigations for therapeutic applications are necessary.

## Figures and Tables

**Table 1:** Total concentration of solids content of fungal extracts and standardization of concentrations to assess antioxidant, antimicrobial, antitumor and toxicity activities.

Fungal Extract	Total Solids Content (%)	Concentration of Fungal Extract Used in Assays (mg mL^−1^)			
		Concentration 1	Concentration 2	Concentration 3	Concentration 4
SXS649	0.97	9.70	4.85	2.42	1.21
SXS650	2.52	25.20	12.60	6.30	3.15
SXS651	2.32	23.20	11.60	5.80	2.90
SXS652	2.70	27.00	13.50	6.75	3.37

**Table 2:** Content of total phenolic compounds and flavonoids in 1 mL of extract of the endophytic fungi *Colletotrichum* sp. SXS649, *Pestalotiopsis* sp. SXS650, Botryosphaeriales SXS651 and *Diaporthe* sp. SXS652 isolates from *P. rigida* Kunth. Values correspond to the mean of the triplicates ± standard deviation.

Fungal Extract	Phenolics (mg of Gallic Acid/1 mL)	Flavonoids (mg of Rutin/1 mL)
SXS649	890 ± 12.50	374 ± 3.00
SXS650	322 ± 6.55	185 ± 1.73
SXS651	233 ± 6.02	84 ± 2.30
SXS652	210 ± 4.61	159 ± 1.52

**Table 3:** IC50 values (mg mL^−1^) of the extracts of the endophytic fungi *Colletotrichum* sp. SXS649, *Pestalotiopsis* sp. SXS650, Botryosphaeriales SXS651, and *Diaporthe* sp. SXS652 isolates from *P. rigida* Kunth that inhibit DPPH free radicals and reduce iron FRAP.

Fungal Extract	IC_50_ DPPH *	AA% **	IC_50_ FRAP ***	Concentration to Achieve IC50 (mg mL^−1^) ****
SXS649	5.6804 ± 0.38	57.8529	8.0917 ± 0.03	4.85
SXS650	22.4748 ± 1.20	51.2709	47.0602 ± 0.03	25.20
SXS651	11.8528 ± 1.06	65.4832	0.00	11.60
SXS652	27.4803 ± 0.00	46.3043	32.2691 ± 0.25	27.00
Control (BHT)	0.0118 ± 0.00	65.5431	-	0.01

Values are the mean of the triplicates ± standard deviation. * Equivalent to IC_50_ calculations (mg mL^−1^) obtained from the graph of absorbance against the concentration for each sample in free radical scavenging. ** Percentage of antioxidant activity calculated by the DPPH method, considering the average concentration of each fungal extract. *** Equivalent to ferrous sulfate (mol Fe^2+^ mg^−1^ of fraction). **** Solid content values of fungal extracts to reach IC_50_ for DPPH and FRAP.

**Table 4:** Viability of *Lactuca sativa* seeds in soil contaminated with 10% soybean oil and treated with extracts (32 mL) of the endophytic fungi *Colletotrichum* sp. SXS649, *Pestalotiopsis* sp. SXS650, Botryosphaeriales SXS651, and *Diaporthe* sp. SXS652 isolates from *P. rigida* Kunth.

Sample Concentrations	Germination (%) (*n* = 240) *	Average Length of Root (mm^2^)	Average Length of the Hypocotyl (mm^2^)
100 g of soil + 32 mL of water (Control 1)	95.00 ± 5.00 ^a^	23.47 ± 2.12 ^a^	41.38 ± 5.47 ^a^
100 g of soil + 10% soy oil (Control 2)	40.00 ± 7.07 ^c^	10.38 ± 2.56 ^b^	18.66 ± 5.35 ^b^
100 g of soil + 10% soy oil + 32 mL of SXS649 fungal extract	72.50 ± 4.33 ^b^	21.36 ± 0.81 ^a^	35.90 ± 1.67 ^a^
100 g of soil + 10% soy oil + 32 mL of SXS650 fungal extract	67.50 ± 4.33 ^b^	20.86 ± 2.96 ^a^	34.49 ± 4.63 ^a^
100 g of soil + 10% soy oil + 32 mL of SXS651 fungal extract	65.00 ± 5.00 ^b^	20.94 ± 1.64 ^a^	36.62 ± 1.19 ^a^
100 g of soil + 10% soy oil + 32 mL of SXS652 fungal extract	60.00 ± 0.00 ^b^	22.87 ± 0.91 ^a^	35.83 ± 4.12 ^a^

Values correspond to the mean of the triplicates ± standard deviation. Values followed by the same letter do not differ statistically by the Tukey test (*p* < 0.05). * (*n* = total number of seeds planted).

**Table 5:** Antagonistic action of the endophytic fungi *Colletotrichum* sp. SXS649, *Pestalotiopsis* sp. SXS650, Botryosphaeriales SXS651, and *Diaporthe* sp. SXS652 isolated from *P. rigida* Kunth against phytopathogenic fungi *A. niger* SXS635, *I. rickii* SXS37, *P. mangiferae* SXS647, and *C. puteana* SXS623 detected by the plate-pairing method and type of competitive interaction, according to the Badalyan scale (2002).

Fungal Taxa	*A. niger*	Inhibition (%) *I. rickii*	*P. mangiferae*	*C. puteana*
SXS649	56.79 ± 0.89 ^a^	50.00 ± 0.51 ^c^	53.16 ± 1.24 ^b^	42.85 ± 1.22 ^b^
	(B)	(A)	(A)	(A)
SXS650	59.25 ± 3.60 ^a^	57.14 ± 1.06 ^a^	48.10 ± 0.66 ^c^	36.73 ± 1.08 ^c^
	(B)	(A)	(A)	(B)
SXS651	54.32 ± 0.87 ^a^	47.14 ± 1.25 ^d^	56.96 ± 0.69 ^a^	38.77 ± 0.89 ^c^
	(A)	(A)	(CA1)	(CA1)
SXS652	50.61 ± 0.87 ^b^	54.28 ± 1.13 ^b^	34.17 ± 0.81 ^d^	76.93 ± 0.81 ^a^
	(B)	(A)	(A)	(CA1)

Values correspond to the mean of the triplicates ± standard deviation. Values with the same lowercase letter do not differ statistically by the Tukey test (*p* < 0.05). (A) “Deadlock” with mycelial contact; (B) distance “deadlock”; (CA1) partial endophytic growth on the phytopathogen after initial deadlock with mycelial contact.

**Table 6:** Toxicity of extracts of endophytic fungi *Colletotrichum* sp. SXS649, *Pestalotiopsis* sp. SXS650, Botryosphaeriales SXS651, and Diaporthe sp. SXS652 isolates of *P. rigida* Kunth against *A. salina* using the Probit dose‒response methodology through the Statplus program. Values correspond to the mean of the triplicates ± standard deviation.

Fungal Extract	CL_50_ (mg mL^−1^)	Break (95% Confidence)	Degree of Toxicity at All Concentrations of Fungal Extract
SXS649	5.00	4.47 ± 5.53	strong
SXS650	12.51	10.25 ± 14.77	strong
SXS651	16.46	15.14 ± 17.77	strong
SXS652	13.23	10.73 ± 15.73	strong
Control (Potassium dichromate)	0.046	0.041 ± 0.51	strong
